# The Oral Ferroportin Inhibitor VIT-2763 Improves Erythropoiesis without Interfering with Iron Chelation Therapy in a Mouse Model of β-Thalassemia

**DOI:** 10.3390/ijms22020873

**Published:** 2021-01-16

**Authors:** Naja Nyffenegger, Anna Flace, Cédric Doucerain, Franz Dürrenberger, Vania Manolova

**Affiliations:** Vifor (International) Ltd., Rechenstrasse 37, 9014 St. Gallen, Switzerland; naja.nyffenegger@viforpharma.com (N.N.); anna.flace@viforpharma.com (A.F.); cedric.doucerain@viforpharma.com (C.D.)

**Keywords:** thalassemia, ineffective erythropoiesis, ferroportin inhibitor, VIT-2763, iron, chelation

## Abstract

In β-thalassemia, ineffective erythropoiesis leads to anemia and systemic iron overload. The management of iron overload by chelation therapy is a standard of care. However, iron chelation does not improve the ineffective erythropoiesis. We recently showed that the oral ferroportin inhibitor VIT-2763 ameliorates anemia and erythropoiesis in the Hbb^th3/+^ mouse model of β-thalassemia. In this study, we investigated whether concurrent use of the iron chelator deferasirox (DFX) and the ferroportin inhibitor VIT-2763 causes any pharmacodynamic interactions in the Hbb^th3/+^ mouse model of β-thalassemia. Mice were treated with VIT-2763 or DFX alone or with the combination of both drugs once daily for three weeks. VIT-2763 alone or in combination with DFX improved anemia and erythropoiesis. VIT-2763 alone decreased serum iron and transferrin saturation (TSAT) but was not able to reduce the liver iron concentration. While DFX alone had no effect on TSAT and erythropoiesis, it significantly reduced the liver iron concentration alone and in the presence of VIT-2763. Our results clearly show that VIT-2763 does not interfere with the iron chelation efficacy of DFX. Furthermore, VIT-2763 retains its beneficial effects on improving ineffective erythropoiesis when combined with DFX in the Hbb^th3/+^ mouse model. In conclusion, co-administration of the oral ferroportin inhibitor VIT-2763 and the iron chelator DFX is feasible and might offer an opportunity to improve both ineffective erythropoiesis and iron overload in β-thalassemia.

## 1. Introduction

β-thalassemia is one of the most common red blood cell (RBC) disorders caused by mutations in the β-globin gene. β-thalassemia is characterized by partially reduced or the complete absence of β-globin synthesis, resulting in an excess of unpaired α-globin chains forming instable aggregates (hemichromes) on the membranes of erythroid precursors and causing their premature death [1,2]. The shortened life span of RBCs leads to anemia and hypoxia, which in turn stimulate erythropoiesis, resulting in increased proliferation and decreased differentiation of erythroid progenitors (ineffective erythropoiesis) in the bone marrow (BM) and extramedullary sites such as the spleen and liver [3]. In addition, anemia and ineffective erythropoiesis in β-thalassemia induce erythropoietin and erythroferrone, which respectively stimulate erythropoiesis and suppress hepcidin. Hepcidin is a liver-derived hormone that binds to ferroportin, the unique iron transporter in mammals, inducing the ubiquitination, internalization, and degradation of ferroportin, thereby blocking iron transport to blood [4,5,6]. 

The current management of anemia in β-thalassemia is by blood transfusions, which cause iron overload, requiring iron chelation. Patients affected by the most severe form of the disease need lifelong blood transfusions (transfusion-dependent thalassemia (TDT)), which results in profound secondary iron overload. Patients with less-severe forms of β-thalassemia, necessitating only occasional or intermittent transfusions, are defined as having non-transfusion-dependent thalassemia (NTDT) but still develop iron overload due to ineffective erythropoiesis and chronic suppression of hepcidin synthesis. Complications of iron overload such as liver cirrhosis and heart failure significantly contribute to morbidity and mortality in β-thalassemia [7]. Hence, the management of iron overload in both TDT and NTDT by chronic iron chelation therapy has become the standard of care. Currently, three iron chelators (deferoxamine (DFO), deferiprone (DFP), and deferasirox (DFX)) are available for the management of iron overload in patients with TDT [8]. DFO is administered parenterally, whereas DFP and DFX are oral drugs with greater dosing convenience. DFX is the only iron chelator tested in controlled clinical studies and approved in the U.S. and Europe for the management of iron overload in patients with NTDT [9].

Despite the important progress in the management of iron overload, iron chelation therapy does not target the underlying disease mechanism in β-thalassemia (i.e., ineffective erythropoiesis). The supplementation of hepcidin mimetics such as minihepcidins, or the induction of endogenous hepcidin synthesis using anti-sense DNA or small interfering RNA oligonucleotides targeting Tmprss6 (*Tmprss6-*ASO or *Tmprss6* siRNA) have been shown to ameliorate ineffective erythropoiesis in the Hbb^th3/+^ mouse model of β-thalassemia intermedia [10,11,12]. Tmprss6 is a membrane-bound serine protease expressed in hepatocytes that negatively modulates hepcidin transcription via the bone morphogenic protein (BMP)/SMAD signaling pathway [13,14,15]. Therefore, inhibition of Tmprss6 expression with anti-sense or RNA interference approaches stimulates hepcidin expression through enhanced BMP signaling. Moreover, combination therapy with the iron chelator DFP and minihepcidins or *Tmprss6*-targeting oligonucleotides improves the ineffective erythropoiesis and diminishes iron overload in the same model of β-thalassemia intermedia [10,16,17]. 

We previously demonstrated that the oral ferroportin inhibitor VIT-2763, similar to hepcidin, induces ferroportin ubiquitination, internalization, and degradation in cell-based assays. In addition, treatment with VIT-2763 ameliorates anemia and ineffective erythropoiesis in the Hbb^th3/+^ mouse model of β-thalassemia [18]. It is anticipated that in clinical settings, VIT-2763 might be co-administered with standard-of-care therapies such as iron chelators. Although all three available iron chelators have proven efficacy in TDT patients, only DFX is currently approved for the management of iron overload in patients with NTDT [8,19,20]. In addition, DFX is administered once daily, providing dosing convenience and a lower pill burden when combined with other oral drugs [20]. Therefore, this study aimed to investigate whether the concurrent use of the iron chelator DFX and the ferroportin inhibitor VIT-2763 causes any pharmacodynamic interactions in the Hbb^th3/+^ mouse model of non-transfusion-dependent β-thalassemia.

## 2. Results and Discussion

### 2.1. VIT-2763 Did Not Impair the Ability of DFX to Reduce the Iron Overload in Hbb^th3/+^ Mice

**Figure 1:**
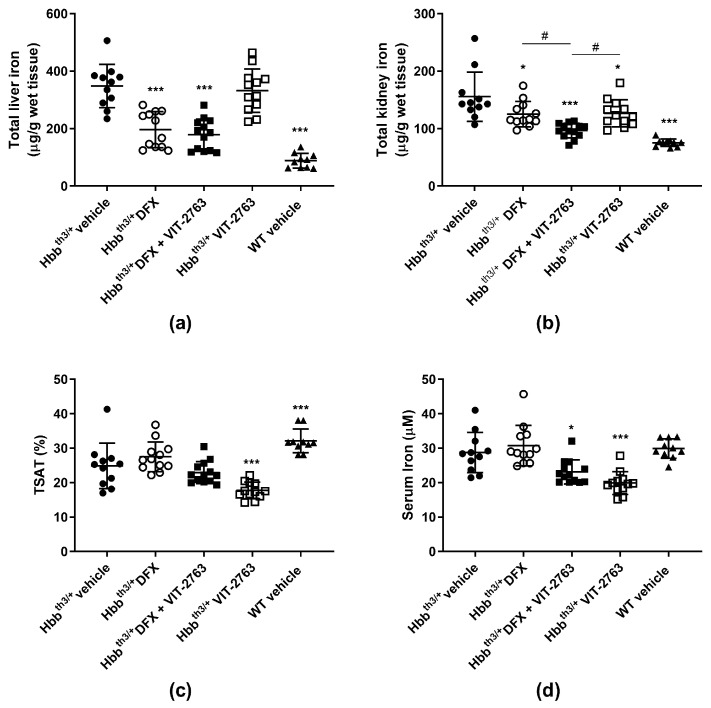
VIT-2763 did not impair the ability of deferasirox (DFX) to reduce iron overload in Hbb^th3/+^ mice. (**a**) The combination of VIT-2763 with DFX resulted in a similar reduction in the liver iron concentration in Hbb^th3/+^ mice as DFX treatment alone. (**b**) Improved kidney iron reduction by the combination of VIT-2763 and DFX. (**c**) Serum iron levels in mice receiving VIT-2763 alone or in combination with DFX. (**d**) Transferrin saturation (TSAT) levels in mice treated with VIT-2763 alone or in combination with DFX. Results represent the mean ± SD. Statistical significance was determined using one-way ANOVA with Dunnett’s multiple-comparison test. * *p* < 0.05 and *** *p* < 0.001. # *p* < 0.05 depicts a comparison to the group treated with the combination of VIT-2763 and DFX. Wild-type (WT) littermates received both vehicles and were included as controls. *n* = 10–13 mice per group.

To investigate whether the concurrent use of VIT-2763 and DFX causes any pharmacodynamic interactions, 11–15-week-old male and female Hbb^th3/+^ mice with established iron overload were dosed per os (p.o.) for three weeks once daily with 30 mg/kg of DFX, 120 mg/kg of VIT-2763, a combination of both compounds, or the vehicle. Total iron concentrations in the livers of Hbb^th3/+^ mice dosed with DFX alone or in combination with VIT-2763 were significantly lower compared to those of vehicle-treated mice (Figure 1a). As expected, VIT-2763 alone did not reverse the established liver iron overload, which reflects its activity to inhibit iron efflux via ferroportin in liver macrophages and hepatocytes. VIT-2763 combined with DFX achieved a similar reduction in the liver iron concentration as DFX alone, suggesting that the reduction of liver iron can be fully attributed to iron chelation. Surprisingly, other groups have shown that the oligonucleotides *Tmprss6*-ASO or *Tmprss6* siRNA, which upregulate endogenous hepcidin transcription, in combination with the iron chelator DFP induce a higher reduction in liver iron compared to DFP alone, despite the lack of effect of the oligonucleotides administered alone on the liver iron concentration in Hbb^th3/+^ mice [16,17]. However, the combination of DFP with minihepcidins lowered the liver iron content in Hbb^th3/+^ mice to similar levels as with DFP alone [10], suggesting a lack of additive effects of iron chelation and iron restriction via this hepcidin mimetic. Interestingly, the combination of DFX and VIT-2763 resulted in a larger reduction in the kidney iron concentration compared to DFX or VIT-2763 alone (Figure 1b; *p* = 0.02 and *p* = 0.01, respectively). This additive effect might be due to iron chelation and excretion mediated by DFX, in addition to the prevention of further iron loading in kidneys by VIT-2763. 

Importantly, efficient iron chelation by DFX in combination with VIT-2763, as shown in Figure 1a, indicates that chelator-induced iron excretion can be achieved despite iron sequestration in liver macrophages and hepatocytes induced by ferroportin inhibition. In addition, previous studies have shown that VIT-2763 prevents de novo liver iron loading in Hbb^th3/+^ mice, most likely by inhibition of dietary iron absorption [18]. Therefore, iron chelation and ferroportin inhibition via VIT-2763 might offer complementary therapeutic effects by lowering the pre-existing iron overload and continuously preventing de novo organ iron accumulation. 

As expected, ferroportin inhibition by VIT-2763 alone significantly reduced serum iron and transferrin saturation (TSAT) levels in Hbb^th3/+^ mice (Figure 1c,d). In Hbb^th3/+^ mice treated with DFX only, TSAT and serum iron levels did not change significantly compared to the vehicle group, and a trend for higher levels was even noted in several independent experiments. This small increase in serum iron levels in DFX-treated mice is most likely due to chelator-bound iron, which is detectable by the assay used to measure serum iron. For the same reason, Hbb^th3/+^ mice treated with the combination of VIT-2763 and DFX showed TSAT and serum iron levels in between those of the single-treatment groups. These data indicate that ferroportin inhibition might be beneficial to prevent an increase in TSAT, which may result in excessive erythroid iron uptake or the generation of noxious non-transferrin-bound iron (NTBI).

### 2.2. Combination of VIT-2763 and DFX Ameliorated Anemia and Ineffective Erythropoiesis in Hbb^th3/+^ Mice

**Figure 2:**
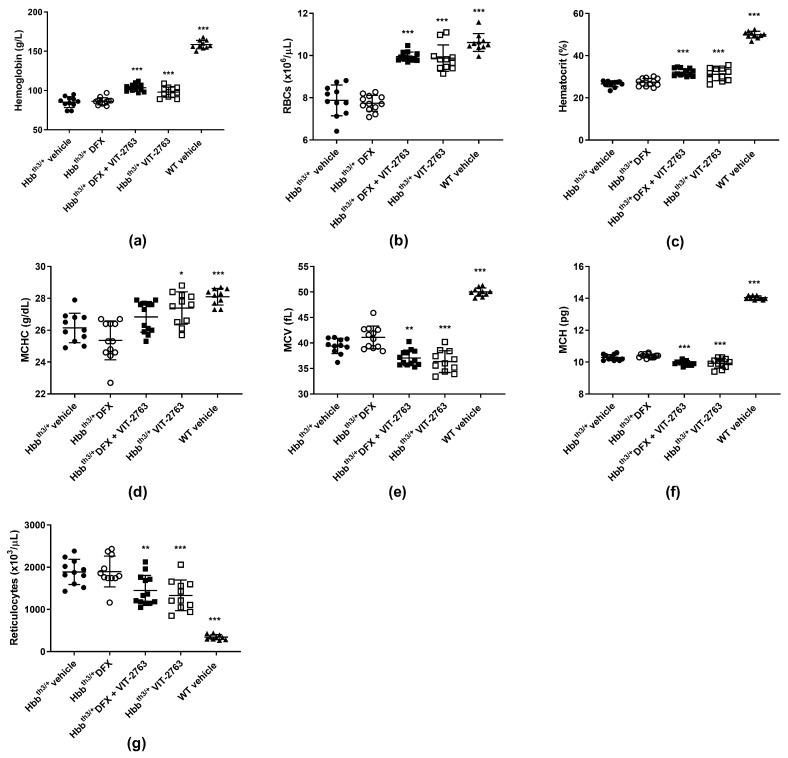
The combination of VIT-2763 and DFX ameliorated anemia and ineffective erythropoiesis in Hbb^th3/+^ mice. VIT-2763 alone or in combination with DFX significantly increased hemoglobin levels (**a**), red blood cell (RBC) counts (**b**), hematocrit (**c**), and mean corpuscular hemoglobin concentration (MCHC) (**d**), and significantly reduced mean corpuscular volume (MCV) (**e**), mean corpuscular hemoglobin (MCH) (**f**), and reticulocyte counts (**g**). DFX alone had no effect. Results represent the mean ± SD. Statistical significance was determined using one-way ANOVA with Dunnett’s multiple-comparison test. * *p* < 0.05, ** *p* < 0.01, and *** *p* < 0.001. Wild-type (WT) littermates received both vehicles and were included as controls. *n* = 10–13 mice per group.

Although DFX alone significantly reduced the liver iron concentration, it did not improve hematological parameters such as hemoglobin, hematocrit, RBC, and reticulocyte counts in the Hbb^th3/+^ model of β-thalassemia (Figure 2a–g). The lack of effect of iron chelation on ineffective erythropoiesis in the Hbb^th3/+^ model has been demonstrated by other groups using the oral iron chelator DFP [10,16,17]. Importantly, VIT-2763 administered alone or concomitantly with DFX significantly ameliorated anemia, observed as increased hemoglobin, RBC, hematocrit, and mean corpuscular hemoglobin concentration (MCHC) and decreased reticulocyte counts. VIT-2763 alone or in combination with DFX decreased the mean corpuscular volume (MCV) and mean corpuscular hemoglobin (MCH), demonstrating iron-restricted erythropoiesis (Figure 2a–g). These data suggest that treatment with an iron chelator alone is not sufficient to improve erythropoiesis, despite the reduced liver iron overload. One possible explanation might be that iron chelators shuttle iron from organs to plasma without reducing the plasma iron pool and consequently the iron uptake by erythroid progenitors. On the contrary, ferroportin inhibition by VIT-2763 causes iron-restricted erythropoiesis (low TSAT), which has been associated with improved erythropoiesis in the Hbb^th3/+^ model of β-thalassemia.

**Figure 3:**
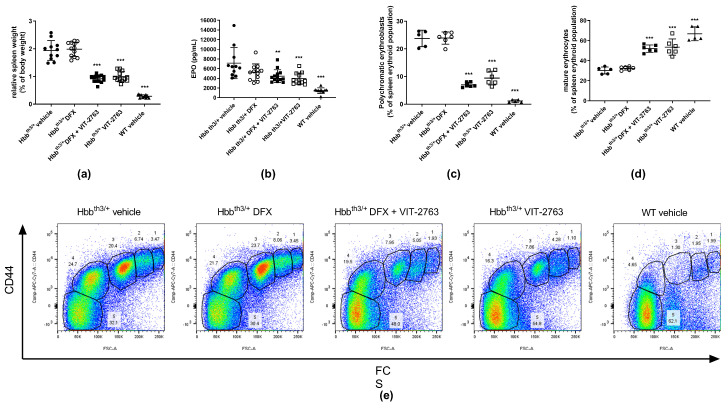
VIT-2763 alone or in combination with DFX improved erythropoiesis in Hbb^th3/+^ mice. (**a**) Splenomegaly was significantly reduced in Hbb^th3/+^ mice treated with VIT-2763 alone or in combination with DFX compared to Hbb^th3/+^ mice treated with vehicle or DFX alone. (**b**) Elevated erythropoietin (EPO) levels were significantly corrected in mice treated with VIT-2763 alone or in combination with DFX. (**c**–**e**) VIT-2763 decreased the frequency of polychromatic erythroblasts (**c**,**e** populations in gate 3) and increased the proportion of mature erythrocytes (**d**,**e** populations in gate 5) in the spleen. Representative dot plots are shown in (**e**). Results represent the mean ± SD. Statistical significance was determined using one-way ANOVA with Dunnett’s multiple-comparison test. ** *p* < 0.01, and *** *p* < 0.001. Wild-type (WT) littermates received both vehicles and were included as controls. (**a**,**b**) *n* = 10–13 mice per group; (**c**–**e**) *n* = 5–6 mice per group.

Plasma erythropoietin (EPO) concentrations in Hbb^th3/+^ mice are elevated in response to anemia and hypoxia induced by the ineffective erythropoiesis. As a result, excessive extramedullary proliferation of erythroid precursors in the spleen leads to splenomegaly. VIT-2763 administered alone or concurrently with DFX significantly reduced the relative spleen weight in Hbb^th3/+^ mice, indicating less extramedullary erythropoiesis. DFX alone had no effect on the relative spleen weight (Figure 3a), further demonstrating that iron chelation does not improve erythropoiesis. Consistent with decreased erythropoietic activity, the serum concentration of EPO declined in mice treated with VIT-2763 alone or in combination with DFX (Figure 3b). Flow cytometry analysis of spleen cells from Hbb^th3/+^ mice stained with Ter119 and CD44 markers showed higher percentages of early erythroid progenitors (gates 1–4) and a lower percentage of mature RBCs (gate 5) when compared to wild-type animals (Figure 3c–e). DFX alone did not change the percentages of erythroid precursors, while VIT-2763 alone or combined with DFX markedly reduced the proportion of immature precursors (gates 1–4), especially polychromatic erythroblasts (gate 3), which decreased by 60% and 70% in Hbb^th3/+^ mice treated with VIT-2763 either alone or in combination with DFX, respectively (Figure 3c,e), compared to Hbb^th3/+^ mice receiving the vehicle. This was associated with a 77% (VIT-2763) and a 72% (DFX + VIT-2763) increase in mature erythrocytes (Figure 3d,e, gate 5) as compared to the Hbb^th3/+^ vehicle group. These data clearly indicate that DFX does not interfere with the therapeutic effects of VIT-2763 to improve ineffective erythropoiesis and ameliorate anemia.

### 2.3. VIT-2763 Alone or in Combination with DFX Improved the Functional Parameters of RBCs in Hbb^th3/+^ Mice

**Figure 4:**
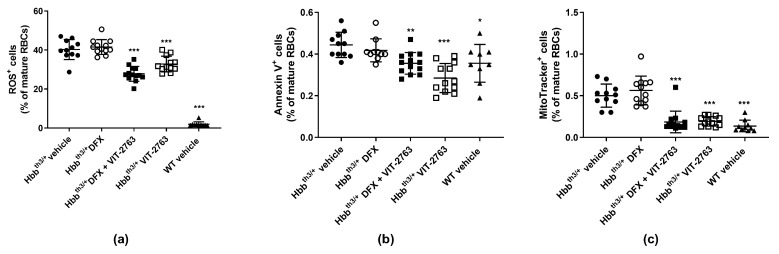
VIT-2763 alone or in combination with DFX improved the functional parameters of RBCs in Hbb^th3/+^ mice. RBCs were gated as mature RBCs (Ter119^hi^CD71^neg^) and analyzed for reactive oxygen species (ROS) (CM-H_2_DCFDA), apoptosis (annexin V as staining for phosphatidylserine (PS)), and retention of mitochondria (MitoTracker Deep Red). VIT-2763 alone or in combination with DFX reduced the percentage of ROS-positive RBCs (**a**), lowered PS exposure on peripheral RBCs (**b**), and improved the elimination of mitochondria in RBCs (**c**) of Hbb^th3/+^ mice compared to Hbb^th3/+^ mice treated with the vehicle or DFX alone. Results represent the mean ± SD. Statistical significance was determined using one-way ANOVA with Dunnett’s multiple-comparison test. * *p* < 0.05, ** *p* < 0.01, and *** *p* < 0.001. Wild-type (WT) littermates received both vehicles and were included as controls. *n* = 9–13 mice per group.

The imbalanced synthesis of α- and β-globin chains of hemoglobin underlying the ineffective erythropoiesis in β-thalassemia leads to the formation of insoluble membrane α-globin aggregates and reactive oxygen species (ROS). Consequently, increased ROS levels cause membrane damage and lead to the exposure of phosphatidylserine (PS) to the extracellular site of the plasma membrane, which serves as an apoptotic signal [21]. VIT-2763 alone or in combination with DFX significantly reduced the percentage of mature RBCs containing intracellular ROS and expressing PS on the outer plasma membrane (Figure 4a,b). Treatment with DFX alone had no effect. These data are in good agreement with published research showing reduced levels of ROS in mature erythroid cells from the BM and spleen of Hbb^th3/+^ mice treated with minihepcidins alone or in combination with DFP, but without the effect of DFP alone [10]. 

Under normal conditions, reticulocytes eliminate mitochondria during maturation through mitophagy, and energy production in mature RBCs is fully dependent on glycolysis [22,23]. In β-thalassemia, mitophagy is impaired and results in the retention of mitochondria in mature RBCs [18,24,25]. Treatment of Hbb^th3/+^ mice with VIT-2763 alone or in combination with DFX significantly decreased the proportion of RBCs containing mitochondria to levels of wild-type mice (Figure 4c). DFX alone showed no effect on the percentage of mitochondria-containing RBCs in Hbb^th3/+^ mice.

### 2.4. VIT-2763 Alone or in Combination with DFX Corrected the Elevated Leukocyte Counts in Hbb^th3/+^ Mice

**Figure 5:**
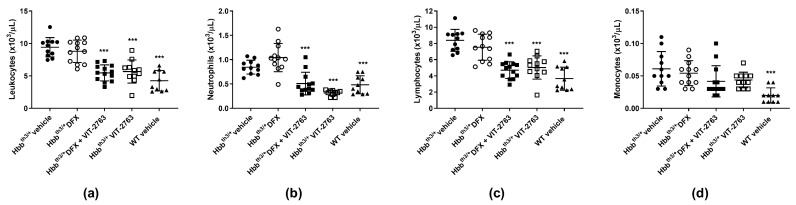
VIT-2763 alone or in combination with DFX corrected pathologically elevated neutrophil counts in Hbb^th3/+^ mice. VIT-2763 alone or in combination with DFX significantly decreased total leukocyte (**a**), neutrophil (**b**), and lymphocyte (**c**) counts, while having no effect on monocytes (**d**). Results represent the mean ± SD. Statistical significance was determined using one-way ANOVA with Dunnett’s multiple-comparison test. and *** *p* < 0.001. Wild-type (WT) littermates received both vehicles and were included as controls. *n* = 10–13 mice per group.

The Hbb^th3/+^ mice showed significantly elevated leukocyte counts compared to wild-type (WT) mice, and dosing with VIT-2763 either alone or in combination with DFX corrected the leukocyte counts close to those of WT mice (Figure 5a). Differential blood counting demonstrated that neutrophils (Figure 5b) contributed mostly to the elevated leukocyte counts, followed by lymphocytes (Figure 5c), whereas monocyte counts did not significantly change (Figure 5d). One possibility is that VIT-2763 improves myelopoiesis in the Hbb^th3/+^ model, as previously demonstrated by our group [18]. Further investigations are necessary to address the molecular mechanism underlying how VIT-2763 either alone or in combination with DFX decreases leukocyte counts in the Hbb^th3/+^ model. Nevertheless, the effect of the ferroportin inhibitor to lower pathologically elevated leukocyte counts suggests that iron restriction by VIT-2763 might have the potential to reduce inflammation in this model of β-thalassemia.

## 3. Conclusions

This study aimed to evaluate whether the concurrent use of the oral iron chelator DFX and the oral ferroportin inhibitor VIT-2763 would lead to pharmacodynamic drug–drug interactions in the Hbb^th3/+^ mouse model of β-thalassemia intermedia. The data presented here show that co-administration of the oral ferroportin inhibitor VIT-2763 and the iron chelator DFX is feasible and might offer an opportunity to improve both erythropoiesis and iron overload in β-thalassemia. Importantly, VIT-2763 in combination with DFX retained its beneficial effects on anemia and ineffective erythropoiesis, as demonstrated by increased hemoglobin levels and RBC counts, reduced reticulocyte counts and spleen size, as well as RBC markers of oxidative stress and apoptosis. This indicates that the iron restriction capacity of VIT-2763 when co-administered with DFX is preserved and the overall available iron for erythropoiesis is still limited, despite iron mobilization from the liver by DFX. In addition, iron chelation efficacy is not negatively impacted despite increased macrophage iron sequestration in VIT-2763-treated mice. One potential advantage of combining VIT-2763 with iron chelators is that ferroportin inhibition on duodenal enterocytes is expected to limit dietary iron absorption in NTDT, which is not feasible by iron chelators alone and might reduce the continuous use of iron chelation therapy or reduce the doses needed. This mechanism of action could be relevant for patients with NTDT, where intestinal absorption due to hepcidin suppression is the key pathogenic mechanism of iron overload. Furthermore, inhibition of ferroportin by VIT-2763 and sequestration of reactive iron (e.g., NTBI) in macrophages may limit potential noxious effects, particularly when the plasma chelator concentration is the lowest between doses. TDT patients on regular blood transfusions and iron chelation therapy have elevated levels of NTBI that correlate with the occurrence of heart disease [26]. Therefore, the sequestration of iron in macrophages by VIT-2763 could further limit the amount of plasma NTBI in TDT patients without interfering with iron chelation. Moreover, in TDT patients on regular blood transfusions, hepcidin levels decrease in the intervals between transfusions, resulting in higher intestinal iron absorption [27]. Hence, it is likely that TDT patients might also benefit from VIT-2763, either alone or combined with an iron chelator, to prevent increased iron absorption between blood transfusions. 

Importantly, our results also give promise that in clinical trials, the efficacy of VIT-2763 can be assessed without interference from the effects of chelation therapy as the current standard of care.

Taken together, this study shows that iron excretion from the liver mediated by DFX can be accomplished despite ferroportin inhibition by VIT-2763. Furthermore, VIT-2763 administered either alone or together with DFX shows similar efficacy in improving anemia, improving erythropoiesis, and normalizing the spleen size of Hbb^th3/+^ mice. Therefore, the combination of iron chelators with VIT-2763 offers the advantage of reversing the established iron overload and improving erythropoiesis in the Hbb^th3/+^ model of β-thalassemia. Based on this preclinical data, we anticipate that patients with β-thalassemia might benefit from both therapeutic modalities without negative pharmacodynamic interaction.

## 4. Materials and Methods

### 4.1. Animal Model

C57BL/6 mice were purchased from Charles River (Sulzfeld, Germany). Hbb^th3/+^ mice (C57BL/6 background) were purchased from Jackson Laboratory [28] and bred under specific-pathogen-free conditions in the animal facility of Vifor (International) Ltd. (St. Gallen, Switzerland), according to the regulations of the Swiss veterinary law. The mice were group-housed, and individual cages were randomly assigned to treatment groups for equal distribution of gender and ages. The animal studies described in this paper complied with all applicable sections of the law and associated guidelines, and were approved by the Veterinary Department of Zurich. All studies were also performed in compliance with the Code of Conduct of the Vifor Pharma Group.

Mice (11–15-week-old male and female) were fed a low-iron diet (LID; Granovit SA, Kaiseraugst, Switzerland; Cat. 2039, Lot 0001901907, Fe = 11 mg/kg) and dosed at 10 mL/kg per os (p.o.) once daily with 30 mg/kg of DFX (Ontario Chemicals Inc., Guelph, ON, Canada), 120 mg/kg of VIT-2763 (Vifor (International) Ltd., St. Gallen, Switzerland), a combination of both compounds (3 h apart), or the respective vehicles. Three hours after dosing, the mice were given access to a standard diet (SD; Granovit SA, Kaiseraugst, Switzerland; Cat. 3437, Fe = approx. 250 mg/kg) for 5–6 h; 0.5% methylcellulose and 30% Kolliphor were used as vehicles for VIT-2763 and DFX, respectively. Dosing was performed in the dark phase of the facility room, corresponding to the active period of rodents. Wild-type (WT) littermates dosed with vehicles served as controls. Hemoglobin levels were determined weekly in tail vein blood using a Hemocue^®^ Hb 201 device.

### 4.2. Analysis of Blood, Serum Iron Parameters, and Tissue Iron

Complete blood counts were analyzed using an Idexx ProCyte analyzer (Idexx Bioresearch, Westbrook, ME, USA). Serum iron levels were determined in triplicate using a MULTIGENT Iron assay (Abbott Diagnostics, Baar, Switzerland). Serum transferrin (*Tf*) was measured in duplicate using mouse-specific ELISA according to the manufacturer’s instructions (Abcam, Cambridge, UK). Transferrin saturation (*TSAT*) was calculated using the following formula:

(1) $$TSAT\;\left(\%\right)=\;\frac{SI\;\left(\left[\frac{\mu g}{dL}\right]\right)}{Tf\left(\;\left[\frac{mg}{dL}\right]\right)}\;\times 71.24$$

Serum EPO was determined in duplicate using mouse-specific DuoSet ELISA according to the manufacturer’s instructions (R&D Systems, Minneapolis, MN, USA). The total liver iron concentration was determined by inductively coupled plasma–optical emission spectroscopy (ICP-OES).

### 4.3. Flow Cytometry for Erythropoiesis, ROS, Mitochondria, and PS Exposure

Erythroid cells from the spleen were analyzed by flow cytometry. Distinct stages of RBC precursors were identified based on the expression levels of Ter119 (allophycocyanine-conjugated rat anti-mouse Ter119, eBioscience, Thermo Fisher Scientific, Basel, Switzerland), CD44 (APC-Cy7-conjugated rat anti-mouse CD44, BioLegend, San Diego, CA, USA), CD71 (Phycoerythrin -conjugated rat anti-mouse CD71, eBioscience, Thermo Fisher Scientific, Basel, Switzerland), and the forward scatter (FSC) as a cell size measure [29]. Mitochondria were detected using MitoTracker Deep Red FM (Invitrogen, Thermo Fisher Scientific, Basel, Switzerland) in RBCs labeled with Ter119 and CD71 antibodies in combination with CM-H_2_DCFDA staining for ROS detection. PS exposure was detected using the Annexin V Apoptosis Detection Kit (Invitrogen, Thermo Fisher Scientific, Basel, Switzerland) on peripheral blood cells labeled with Ter119 and CD71 antibodies.

### 4.4. Statistical Analysis

For analysis of endpoint parameters, one-way ANOVA with Dunnett’s multiple-comparison test was used. Data are presented by individual value with the mean as scatter plots. Significant differences between treatment groups compared to the Hbb^th3/+^ vehicle group are indicated: * *p* < 0.05, ** *p* < 0.01, and *** *p* < 0.001.

## Data Availability

Not applicable.
